# Complement Components in the Diagnosis and Treatment after Kidney Transplantation—Is There a Missing Link?

**DOI:** 10.3390/biom11060773

**Published:** 2021-05-21

**Authors:** Małgorzata Kielar, Agnieszka Gala-Błądzińska, Paulina Dumnicka, Piotr Ceranowicz, Maria Kapusta, Beata Naumnik, Grzegorz Kubiak, Marek Kuźniewski, Beata Kuśnierz-Cabala

**Affiliations:** 1 St. Louis Regional Children’s Hospital, Medical Diagnostic Laboratory with a Bacteriology Laboratory, Strzelecka 2 St., 31-503 Kraków, Poland; gkielar@tlen.pl; 2 Medical College of Rzeszów University, Institute of Medical Sciences, Kopisto 2A Avn., 35-310 Rzeszów, Poland; agala.edu@gmail.com; 3 Jagiellonian University Medical College, Faculty of Pharmacy, Department of Medical Diagnostics, Medyczna 9 St., 30-688 Kraków, Poland; paulina.dumnicka@uj.edu.pl; 4 Jagiellonian University Medical College, Faculty of Medicine, Department of Physiology, Grzegórzecka 16 St., 31-531 Kraków, Poland; piotr.ceranowicz@uj.edu.pl; 5 Jagiellonian University Medical College, Faculty of Medicine, Chair of Clinical Biochemistry, Department of Diagnostics, Kopernika 15A St., 31-501 Kraków, Poland; mbmkapus@cyf-kr.edu.pl; 6 Medical University of Białystok, Faculty of Medicine, 1st Department of Nephrology and Transplantation with Dialysis Unit, Żurawia 14 St., 15-540 Białystok, Poland; bnaumnik@poczta.onet.pl; 7 Catholic University of Leuven, Department of Cardiovascular Diseases, 3000 Leuven, Belgium; grzegorz.kubiak@uzleuven.be; 8 Jagiellonian University Medical College, Faculty of Medicine, Chair and Department of Nephrology, Jakubowskiego 2 St., 30-688 Kraków, Poland; marek.kuzniewski@uj.edu.pl

**Keywords:** kidney transplantation, allograft function, complement, transplant rejection, complotypes

## Abstract

Currently, kidney transplantation is widely accepted as the renal replacement therapy allowing for the best quality of life and longest survival of patients developing end-stage renal disease. However, chronic transplant rejection, recurrence of previous kidney disease or newly acquired conditions, or immunosuppressive drug toxicity often lead to a deterioration of kidney allograft function over time. Complement components play an important role in the pathogenesis of kidney allograft impairment. Most studies on the role of complement in kidney graft function focus on humoral rejection; however, complement has also been associated with cell mediated rejection, post-transplant thrombotic microangiopathy, the recurrence of several glomerulopathies in the transplanted kidney, and transplant tolerance. Better understanding of the complement involvement in the transplanted kidney damage has led to the development of novel therapies that inhibit complement components and improve graft survival. The analysis of functional complotypes, based on the genotype of both graft recipient and donor, may become a valuable tool for assessing the risk of acute transplant rejection. The review summarizes current knowledge on the pathomechanisms of complement activation following kidney transplantation and the resulting diagnostic and therapeutic possibilities.

## 1. Introduction

Kidney transplantation is a widely used method for treatment of end-stage renal failure (RF) providing improved survival and quality of life. Kidney transplantation is the best renal replacement therapy, capable of resolving all uremic symptoms as long as the allograft function remains well preserved. However, despite the progress in the donor-recipient matching, transplant rejection remains a challenge [1,2].

In the monitoring of allograft function, eGFR based on serum creatinine remains the method of choice, accompanied by other laboratory tests, such as proteinuria and urinalysis, and by kidney ultrasound imaging [3,4]. However, these classical biomarkers are neither sensitive enough to exclude the possibility of chronic kidney transplant rejection nor specific enough to differentiate chronic rejection from other causes of decreasing graft function. Therefore, in clinical practice, the assessment of allograft rejection is not easy.

Kidney biopsy is currently the gold standard in assessing the causes of a deterioration of allograft function [3,4]. An experienced pathologist evaluates the bioptate using specialized staining techniques and light, immunofluorescence, and electron microscopy. One possible abnormality found during histopathological examination is the presence of C4d complement deposits in the peritubular capillaries [1,5]. C4d is a degradation product of the complement factor C4 resulting from complement classical pathway activation. C4d deposition indicates humoral rejection of the allograft. However, there are controversies regarding the sensitivity of this test: the estimates vary significantly from 23% to 95% [1]. Moreover, a variety of conditions may coexist in the transplanted kidney that would make it difficult to interpret the overall clinical and morphological picture.

Our aim was to review the impact of complement on kidney allograft function and to summarize the knowledge about the impact of emerging therapies on complement activation in renal transplant recipients.

## 2. The Role of Complement in Human Immunity

### 2.1. Systemic Roles of Complement

**Figure 1:**
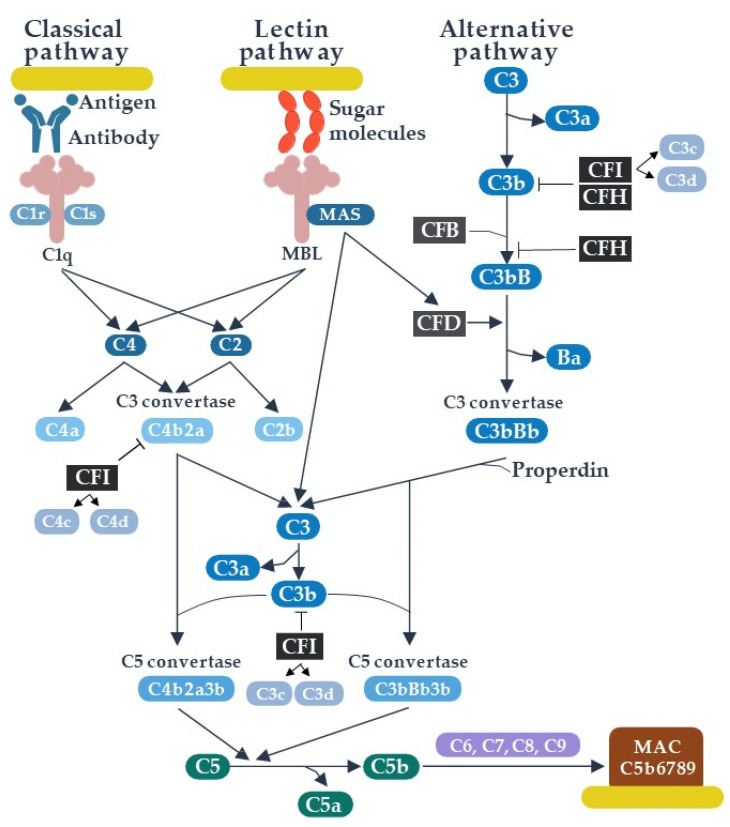
Pathways of complement activation. CFB—complement factor B, CFD—complement factor D, CFH—complement factor H, CFI—complement factor I, MASP—MBL associated serine proteinase, MAC—membrane attack complex. CFI inhibits C3b and C4b and degrades them into fragments (complement split products); of those, C3d and C4d remain bound to the target cells and may be detected to confirm complement activation.

The complement system plays an important role as a link between the innate and the adaptive immune responses, supporting the processes of chemotaxis, opsonization, phagocytosis, and cell lysis of pathogens, as well as the removal of immune complexes and apoptotic cells. As a part of the innate immunity, the complement factors are specialized in recognizing and controlling pathogens; however, complement components are also involved in hemostasis, apoptosis, tumor pathogenesis, and autoimmune diseases [6]. As a part of adaptive immunity, complement is activated by immune complexes while complement components may stimulate activation of B and T-cells [7]. Following organ transplantation, complement activation enhances immune responses to alloantigens [6,8]. The complement system consists of about 50 proteins, mainly dissolved in plasma and body fluids, including zymogens and regulatory proteins that control the activation process and prevent excessive activation. The receptors for the active components of the complement are exposed by most immune cells [9,10,11]. The activation of the complement system occurs in an enzymatic cascade, triggered in three distinct pathways of activation (classical, alternative and lectin), leading to a common final stage that is the creation of a membrane attack complex (MAC) capable of inducing direct cell lysis (Figure 1) [12].

The classical complement pathway is activated by binding of C1q component with immunoglobulins M (IgM) or IgG complexed with antigens; thus, it is a part of acquired immunity [13]. The lectin pathway, recognized as an important mechanism of acute phase response to infection, begins with the binding of mannan-binding lectin (MBL), ficolins or collectins (the pattern recognition proteins) to pathogen-associated molecular patterns such as D-mannose present on the surface of pathogens [14]. The alternative complement activation pathway is a part of the innate immune system and is constantly activated in plasma due to instability of the C3 component that undergoes continuous hydrolysis. The C3b fragment may bind to bacteria, yeast, virus-infected or damaged cells, C-reactive protein, or polysaccharides; otherwise, it is inactivated by regulatory proteins. Except for the three widely recognized activation pathways, serine proteases involved in coagulation, mainly thrombin, have been shown to activate C3 fragment [12,13].

Further steps of complement activation are common and include the breakdown of the C3 component into active products (C3a and C3b) and the formation of C5 convertase, which cleaves the C5 protein. C3a and C5a fragments serve as anaphylatoxins, while the resulting C5b molecule binds to C6, C7, C8, and C9 proteins forming a membrane attack complex (MAC), responsible for target cell’s lysis [12,13] (Figure 1).

Complement activation is tightly controlled by a wide range of inhibitors, preventing damage to own cells, working at various stages of complement activation. These include membrane proteins inhibiting the formation and action of C3 convertase (e.g., membrane cofactor protein—MCP, decay-accelerating factor—DAF, or CD55) and further stages of complement activation (CD59, vibronectin) as well as soluble proteins (C1q inhibitor, factor H, factor I, C4-binding protein, and carboxypeptidase N) [12,13].

### 2.2. Local and Intracellular Roles of Complement

Although complement has long been viewed as a system of plasma proteins mainly produced in liver, it must be noted that complement components may also be produced locally, e.g., in renal tubular epithelium [15]. Notably, kidney tubular cells can synthesize most complement components [16]. Moreover, recent discoveries brought the evidence of intracellular synthesis of C3a and C5a in T-cells, vital for their survival and activation [17,18]. Intracellular activation of C3 by cathepsin L and binding of C3a with its receptor (C3aR) present on lysosomes induces low level activation of mammalian target of rapamycin (mTOR) required for survival of CD4+ T-cells. As a part of T-cell activation, intracellularly generated C3a and C3b fragments are transported to the cell membrane and act as the autocrine stimulants (via membrane C3aR and MCP), vital for the generation of interferon-γ (IFN-γ) [19]. Intracellular C5a–C5aR1 interaction is required for interleukin-2 synthesis and T helper cells type 1 (Th1) activation, while the C5a—C5aR2 interaction is necessary for interleukin 10 synthesis and Th1 contraction [19].

## 3. The Role of Complement in Kidney Graft Injury

### 3.1. Ischemia-Reperfusion Injury of Kidney Graft

During ischemia of kidney graft, the cells of the transplant are deprived of both oxygen and nutrients, which disrupts the cells’ metabolism leading to necrosis. The quality of the donor organ and the duration of ischemia are known to affect the long-term survival of the kidney allograft. Kidneys from living donors present better function and longer survival than those obtained from deceased donors following brain death or cardiac death [20]. Damman et al. [10] demonstrated higher C3 gene expression and increased C3d deposits in the biopsies of kidneys obtained from brain-dead donors as compared to living donors. Hence, complement activation in the kidney during cold ischemia may affect the function of the renal allograft after transplantation. More recent results of Damman et al. [21] indicate that the activation of complement (and coagulation cascade) initiates in donor kidneys following brain death and before the retrieval of the kidneys from the donor.

The subsequent reperfusion exerts further damage to the organ. When circulation is restored to the transplanted organ, necrotic and apoptotic cells activate the innate immune response by stimulating leukocytes and macrophage migration to inflammation sites. The proinflammatory cytokines, chemokines and reactive oxygen species play a key role in exacerbating the immune and inflammatory response. As ischemia-reperfusion injury (IRI) progresses, damage-associated molecular patterns (DAMPs) are generated that perpetuate the cellular inflammatory response and enable the activation of complement activation pathways [22,23]. Pratt et al. [15] showed that C3 activated during transplant reperfusion is a triggering factor of IRI and is associated with late allograft damage and rejection. Moreover, as shown by studies on mice with complement deficiencies [24], C3 and C5 components play a significant role in IRI. The deficiency of C3, C5, and C6 (but not C4) components in mice was protective against IRI in kidney allograft [25,26].

C5a and C3a anaphylatoxins increase the inflammatory response. Formed by proteolytic degradation, C5a fragment serves as a chemotactic agent for neutrophils and macrophages, there is also evidence that C3a induced chemotaxis of monocytes and mast cells [27]. The C5a fragment can bind to two types of receptors on macrophages and neutrophils: C5aR1 and C5aR2 (the latter also binding C3a), initiating a number of effector processes (chemotaxis, activation of mitogen-dependent protein kinases, increase in intracellular calcium ion concentration, and intracellular degranulation) [28]. C5a/C5aR signaling has been identified as a profibrotic pathway in the kidney [29]. 

The ischemia-reperfusion injury induces the above mechanisms of innate immunity; however, it also enhances antigen presentation and stimulates B and T-cell mediated adaptive immunity. The inflammatory milieu associated with complement activation in transplanted organ supports the maturation of antigen-presenting cells. The complement receptor 2 signaling increase B lymphocytes response to antigens present on C3d-opsonized cells [14]. Complement receptors 2–4 are present on myeloid cells, including B and T-cells, and their activation by C3b fragments may increase cytokine production and phagocytosis [14,30]. 

Ischemia-reperfusion injury has been associated with the activation of all three complement pathways [14,30]. The C3 split products (C3c and C3d) detected in kidney biopsies serve as a proof of complement activation regardless of the pathway. In the study of Bobka et al. [31], several complement components, including C3c and C3d, were detected already in “zero-biopsies” taken at the time of transplantation, before implantation of the kidney. The study included 44 patients. C3c was detected in glomeruli (mesangial matrix and basement membrane), tubular cells (weak staining), and peritubular endothelial cells. The staining was higher in kidneys transplanted to patients who developed complications (delayed graft function or antibody-mediated or T-cell mediated rejection during subsequent year); however, low staining was detected also in controls without complications during the one-year follow-up [31]. 

### 3.2. Humoral Rejection of Kidney Allograft

Antibody-mediated rejection (AMR) is currently recognized as a leading cause of late or chronic transplant rejection [32,33,34,35]. Moreover, it is estimated that 30–50% of acute rejection episodes occur due to AMR [35]. The recipient’s alloantibodies bind the antigens exposed on the endothelial cells of the graft. The donor-specific antibodies (DSAs) may be present in a recipient at the time of transplantation (preformed), or their production may be induced de novo after transplantation. The immune complexes formed on the surface of kidney allograft endothelium bind C1q complement component, leading to the activation of the classical complement pathway. The C4d fragment formed following this activation, and deposited in the peritubular capillaries of the kidney allograft, is an important biomarker of antibody-mediated rejection [5]. Complement activation leads to inflammation and thrombosis in the graft microcirculation, resulting in ischemia, apoptosis, necrosis, and consequently the allograft failure [36].

Sensitive methods for identifying anti-human leukocyte antigens (HLA) antibodies and DSA, as well as the methods for determining the C4d component in kidney tissue collected during protocol or diagnostic biopsy allow the confirmation of AMR. The C4d deposits in peritubular capillaries, are included in the Banff classification [5,37] among the criteria allowing for the differentiation between acute and chronic cellular and humoral transplant rejection. The diagnosis and classification of antibody-mediated rejection is based on serology (detection of circulating DSA directed to HLA or non-HLA antigens), as well as on kidney biopsy examination including immunomorphology (the presence of linear C4d deposits in peritubular capillaries in active AMR) and morphology (microvascular inflammation in glomeruli and peritubular capillaries, arteritis in active cases, transplant glomerulopathy or peritubular capillary basement membrane multilayering in chronic cases) [37]. The assessment of C4d deposits is mandatory in the transplanted kidney’s biopsy using both immunofluorescence (frozen tissue) and immunohistochemistry (paraffin-embedded tissue). Despite the high specificity of the C4d test, it is not a marker of high diagnostic sensitivity. The studies assessing the expression profiles of transplant endothelial cells revealed that only about 40% of kidney transplants with histopathological morphological features compatible with antibody-mediated rejection and simultaneously increased endothelial gene expression indicating endothelial injury were C4d-positive [32,38,39]. The addition of alloantibodies to endothelial cells cultured in vitro have been shown to induce expression of inflammatory mediators in the absence of complement [38,40]. Moreover, endothelial cells may be damaged by natural killer cells or macrophages that recognize the endothelial cell-bound alloantibodies through the Fc receptor [38]. 

Recently, the ability of DSA to bind C1q complement component has been proposed as a biomarker of AMR [41,42]. Binding of C1q is a first step in the activation of classical complement pathway; thus, the ability to fix C1q indicates the cytotoxic potential of the antibodies. In the study of 1016 patients after kidney transplantation, Loupy et al. [36] confirmed the usefulness of determining complement-binding donor-specific anti-HLA antibodies in the diagnosis and risk assessment of transplant rejection. The presence of C1q-binding DSA was associated with the presence of C4d deposits in peritubular capillaries detected in an allograft biopsy. The detection of C1q-binding DSA permitted the detection of a humoral transplant rejection in C4d-negative subjects; as noted by Loupy et al. [36], a rejected allograft may present a pattern of C4d-/C1q-DSA+. Furthermore, Viglietti et al. [43] reported increased incidence of graft loss among kidney transplant recipients with C1q-fixing anti-HLA DSA detected following the episode of acute AMR. Of interest, despite treatment of AMR with plasma exchange, intravenous immunoglobulin and rituximab, complement-binding DSA remained detectable in about one-third of patients who tested positive at the diagnosis of AMR, and notably, there were patients with non-complement binding DSA at diagnosis who developed complement-binding DSA during three months post-AMR treatment [43]. C1q-fixing ability was associated with higher concentrations of DSA, expressed in mean fluorescence activity [43]. Possibly, C1q-fixing DSA included in the diagnostic protocol may serve in the future as an early marker of AMR risk, allowing early modification of therapy and optimization of immunosuppression. 

In turn, in the study of Sicard et al. [44], detection of the presence of C3d-binding anti-HLA DSA was a more sensitive and specific indicator of early transplant damage. In the study of 69 patients, the diagnostic sensitivity to detect AMR was compared by examining three parameters: the C4d deposits in peritubular capillaries, the presence of C1q-binding DSA, and the C3d-binding DSA. At one year and three years after transplantation, detection of C3d-DSA had a higher diagnostic sensitivity and specificity compared to the other two tests.

The advanced age of the kidney graft recipient is associated with a reduction in the humoral response and a lower risk of developing DSA [45]. Perhaps the assessment of complement components-fixing DSA in the population of senior recipients would provide the opportunity to adapt an immunosuppressive therapy adequately to the immune status of these patients.

Antibodies other than anti-HLA may also be responsible for both acute and chronic humoral rejection. In cases of the presence of C4d deposits in a transplanted kidney and the absence of anti-HLA in the blood, the possibility of complement activation by other antibodies should be considered [46,47].

### 3.3. Cellular Rejection of Kidney Allograft

Recognition of foreign antigens by a recipient’s T-cells is the first step that leads to the activation, proliferation, and differentiation of alloreactive T-cells and T-cell mediated rejection. The recipients’ antigen presenting cells (APCs), including dendritic cells, macrophages, and B lymphocytes internalize the antigen, process and hydrolyze it, and expose the epitopes on the cell surface in a complex with HLA class II molecules. APCs are also able to present the exogenous antigens with HLA class I in a process called cross-presentation, which is crucial for activating CD8+ cytotoxic T lymphocytes. Moreover, the donor’s HLA molecules (both class I and II) present on the donor’s dendritic cells may be directly recognized by the recipient’s T cells (reviewed in [48]). The presented antigens stimulate CD4+ or CD8+ T-cells via a T-cell receptor (TCR)–CD3 complex. To activate T-cells, simultaneous costimulatory signals are required, including interactions of B7 proteins present on the surface of APCs with CD28 on T-cell or CD40 with CD40 ligand [49]. The resulting secretion of interleukin 2 (IL-2) activates both helper (Th) and cytotoxic (Tc) T lymphocytes. CD4+ Th lymphokines increase the permeability of blood vessels and stimulate local accumulation of cytotoxic T-cells. The released perforins destroy cell membranes of the target graft cells, while granzymes degrade mitochondria, digest histones and activate DNAses. This ultimately leads to apoptosis of allograft cells. The second mechanism triggering programmed cell death (apoptosis) is the activation of Fas ligand on T-cells, which binds to Fas (CD95) on target cells [50]. 

In delayed-type hypersensitivity, macrophages serve as the main effector cells. An increased production of tumor necrosis factor α (TNFα) and interferon α (IFNα) by activated Th1 lymphocytes leads to increased permeability of blood vessels, swelling of the allograft and increased infiltration by T-cells, macrophages, and neutrophils. Nitric oxide produced by activated inducible nitric oxide synthase (iNOS) exacerbates this effect. TNFα, when combined with its specific receptor on the target cell, initiates the apoptotic process by activating the caspase pathway (protein hydrolysis, degradation of genetic material) [51].

Recent studies highlight the important role of complement as a key modulator of T-cell-mediated alloimmunity. Local production of C3a and C5a by APC is necessary for T-cell co-stimulation [52,53]. C3a and C5a are produced by T-cells and exert autocrine effects necessary for proper stimulation of Th1 lymphocytes [19]. Properdin and factor H production by dendritic cells modulate T-cells [54]. Animal studies have shown that a deficiency in the recipient’s C3 complement component prevents induction of chemokines and cytokines and weakens the priming, expansion, and infiltration of heart transplants by donor-reactive T-cells in response to IRI. These new findings link IRI damage to T-cell rejection through MBL-induced complement activation [24]. On the other hand, complement components including C1q, C3, C4, or C5a have been assigned immunomodulatory effects on APCs, associated with induction of immature tolerogenic phenotypes of dendritic cells [8].

### 3.4. Post-Transplant Thrombotic Microangiopathy (TMA)

Thrombotic microangiopathy is a syndrome that includes microvascular thrombosis, thrombocytopenia, hemolytic anemia, and red blood cell fragmentation. In kidney recipients, this disease is not uncommon and often occurs without systemic symptoms, with TMA features detectable solely in a biopsy of the transplanted kidney [55,56]. TMA may arise de novo in the kidney transplant or may be the recurrence of the underlying disease. The most common causes of recurrent TMA are mutations in the genes encoding the complement components: *CFH*, *CFI*, *MCP*, and *C3*, associated with atypical hemolytic uremic syndrome (aHUS). Several complement haplotypes increase the risk of TMA in various populations. In transplant recipients, TMA may be the result of complement activation triggered by concomitant autoimmune diseases, malignant hypertension, infections (in particular with hepatitis C virus), and antibody mediated rejection [57]. Additionally, TMA may develop following endothelial cell damage induced by treatment with calcineurin or m-TOR inhibitors. Although higher doses or the combination of calcineurin inhibitors and m-TOR inhibitors have been associated with higher incidence of TMA after kidney transplantation [58,59], the complication occurs in a small proportion of patients treated with these drugs, implicating additional predisposing factors [56]. Interestingly, in a small series (24 patients) of de novo TMA after kidney transplantation, *CFH* and *CFI* mutations compatible with aHUS have been identified in 29% of patients [60]. However, the causes of TMA after renal transplantation may be complex. In a recent retrospective study of Broecker et al. [61], calcineurin inhibitor treatment or antibody-mediated rejection were identified as the most common causes of TMA (in 22% and 11% of patients, respectively); however, the etiology was uncertain or unknown in 63% of patients. In 56% of patients, one or more underlying factors were identified as a possible cause or trigger of TMA, including prothrombotic conditions (e.g., antiphospholipid syndrome), malignant hypertension, treatment for tuberculosis, de novo post-infectious glomerulonephritis, acute cytomegalovirus infection, lung transplantation, pancreatic surgery, sepsis, and histiocytic glomerulopathy [61].

Knowledge of TMA pathomechanisms based on complement activation after kidney transplantation requires extensive diagnosis of possible causes of this disease. In some cases, preventative treatment is an option for causal TMA, discussed later in the manuscript. 

### 3.5. Recurrent Nephropathy in a Transplanted Kidney

Activation of complement in a transplanted kidney may also be associated with the recurrence of a disease that has damaged the patient’s own kidneys. Mutations in genes encoding soluble complement regulatory proteins such as factor H, factor H-related proteins 1–3 and 5 and factor I as well as activating proteins such as component C3 and factor B may cause the abnormal activation of the alternative complement pathway in the transplanted kidney. Activation of the alternative complement pathway causes glomerular damage and recurrence of glomerulopathy in allograft that may be associated with TMA [57,62]. The rare glomerulopathy caused by the defective regulation of the alternative complement pathway and characterized by C3 deposition in the glomeruli (detected by immunofluorescence) in the absence of immunoglobulin/immune complexes has been defined as C3 glomerulopathy [63]. After transplantation, the recurrence of C3 glomerulopathy is commonly observed (in about 70% of patients) [64].

In immune complex associated membranoproliferative glomerulonephritis, complement activation seems induced by the formation of immune complexes. The recurrence of membranoproliferative glomerulonephritis associated with polyclonal immunoglobulin deposits in the kidney allograft is less common as compared to C3 glomerulopathy, and the lack of C3 or C4d deposits is associated with lower rate of recurrence [65]. Moreover, it has been shown that mutations in complement regulating and activating genes are responsible for the severity of glomerulonephritis [66,67].

### 3.6. Calcineurin Inhibitor-Induced Nephrotoxicity

Acute calcineurin inhibitor nephrotoxicity is dose-dependent and reversible after dose reduction. It occurs early after initiation of treatment and has been associated with vasoconstriction of the afferent and efferent glomerular arterioles, endothelial dysfunction, and resulting reduction in renal blood flow [68]. The association of calcineurin inhibitors with thrombotic microangiopathy has been reviewed above. Chronic calcineurin inhibitor-induced nephrotoxicity was long believed to be an important cause of late graft failure; however, newer studies underscore the impact of chronic antibody-mediated rejection (which may actually be associated with non-compliance and low immunosuppressive drug concentrations) [68]. The histopathologic features attributed to chronic calcineurin inhibitor nephrotoxicity (arteriolar hyalinosis, interstitial fibrosis and tubular atrophy, focal segmental or global glomerular sclerosis) are not specific, and the mechanisms underlying these changes are not clear [68]. 

Some animal and in vitro experimental studies indicate the involvement of complement activation in the pathogenesis of calcineurin inhibitor-induced nephrotoxicity. Treatment of mice with subcutaneous cyclosporin A induced tubular injury and interstitial fibrosis associated with increased deposition of C4d, C3 in renal tubular epithelium and MAC component (C9) in the interstitium and renal proximal tubules [69]. In vitro, cyclosporin A has been shown to induce the release of complement activating microparticles from endothelial cells. Similar microparticles have been detected in blood from kidney transplant recipients [70]. In the study of Renner et al. [70], cyclosporin A induced microparticles increased activation of alternative complement pathway and were associated with endothelial injury in vitro. Moreover, injection of such microparticles into the blood of experimental animals (mice) induced local mesangial activation of complement and mesangial proliferation. In addition, calcineurin inhibitors have been shown to induce complement activation (including formation of MAC) and decrease expression of complement regulatory proteins in cultured human renal tubular cells [71,72]. 

## 4. Therapies Affecting Complement in Kidney Transplantation

Bearing in mind the important role of complement in the processes of IRI, kidney transplant rejection and tolerance to allograft, novel drugs are being investigated for effectively targeting the complement components. At present, polyclonal antibodies (human intravenous immunoglobulin, IVIG) are used in standard treatment regimens in active AMR, which inhibit complement activation by blocking the receptor for the Fc fragment of immunoglobulin (FcR) or the C1q component [73,74,75].

Rituximab has been recently proposed as an adjunctive therapy in acute AMR; however, the evidence is based mainly on observational studies [73,76]. Rituximab is a chimeric human-mouse monoclonal antibody, a glycosylated immunoglobulin containing human IgG1 constant sequences and mouse light and heavy chain sequences. The drug selectively binds to the transmembrane antigen CD20 found on the surface of B lymphocytes which leads to cell death (due to T-cell and complement-mediated effects and apoptosis); some authors also underscore the inhibitory effect on the production of complement-inducing antibodies [75,77]. It is routinely used in post-transplant lymphoproliferative disorders. It is increasingly used in desensitization protocols in AB0-incompatible graft recipients or highly immunized recipients [78,79,80] (Table 1). To the contrary, the proteasome inhibitor bortezomib did not prove to be efficient in late antibody-mediated rejection in kidney transplant recipients [81].

Inhibition of the C5 complement component is a new therapeutic strategy that may reduce the incidence of AMR in highly immunized transplant recipients. This effect can be obtained by using the anti-C5 monoclonal antibody eculizumab. The drug is registered for the treatment of paroxysmal nocturnal hemoglobinuria and aHUS. In transplantation, eculizumab is not routinely administered. However, Stegall et al. [85] showed the efficacy of eculizumab treatment in prevention of early AMR among sensitized recipients of kidney grafts from living donors (Table 2), which was further confirmed by Marks et al. [70]. Blocking the effector activity of complement with anti-C5 monoclonal antibody eculizumab is the treatment of choice in cases of recurrent TMA after kidney transplantation [86]. Moreover, many centers use eculizumab off-label, for treating de novo TMA. Large randomized, prospective studies using anti-C5 in TMA are still lacking [87,88,89,90].

Another drug, registered for the treatment of angioedema type I and II, a C1 esterase inhibitor (C1-INH), is being trialed in patients after transplantation. This is a glycoprotein with a molecular weight of 105 kDa belonging to the human plasma system of serine protease inhibitors (serpins). Under physiological conditions, it blocks the classical pathway of complement activation by inactivating the C1s and C1r components. In hereditary angioedema, C1-INH works by substituting the missing C1 esterase inhibitor activity. The C1 inhibitor can irreversibly block C1 components [92] as well as MASP-1 and MASP-2 [93]. Therefore, it is an effective inhibitor of the activation of both the classical and lectin complement pathways. The safety of the C1-INH was evaluated in highly immunized kidney transplant recipients, and the treatment resulted in the reduction in C1q+ HLA antibody concentration and the diminished occurrence of humoral rejection [75]. Studies in kidney transplant recipients are limited (Table 3); however, the results regarding AMR treatment and prevention of delayed graft function are encouraging and no serious toxicity was observed [94].

Currently, clinical trials for using other substances that act on complement components in preventing kidney transplant rejection are underway [95,96,97]. The soluble form of complement type 1 receptor (CR1; CD35) has shown promising therapeutic effects. This protein, which is a natural membrane regulator of complement, inhibits both the classical and alternative complement activation pathways. Recombinant soluble CR1, a C3 convertase inhibitor (APT070, mirococept), used in perfusion of a kidney’s allograft in rodents, has been shown to reduce IRI-associated kidney damage, effectively preventing acute renal tubular damage, and prolonging kidney allograft survival [98].

The studies of inherited sets of polymorphisms in the genes of complement proteins and regulatory proteins (the complotypes) have the potential to open future diagnostic options affecting therapeutic decisions in patients after organ transplantation [99,100].

## 5. The Role of Complement in Transplant Tolerance

To prevent rejection of the transplanted organ and extend the survival of the graft, it is necessary to use immunosuppressive therapy with minimal adverse effects. The average survival time for a kidney transplant has not increased significantly in recent years. Hence, transplant tolerance remains the focus of both scientists and clinicians.

CD4(+) Foxp3(+) regulatory T-cells (Treg) are the main regulators of immune homeostasis and immune tolerance. According to the classic concept, complement counteracts the invasion of foreign antigens through opsonization or by direct killing of the alloantigen-bearing cells. The activation of complement components leads to inflammation and activation of the acquired immune response at various levels. Nevertheless, in recent years it has become evident that, under certain conditions, complement may also directly or indirectly induce a pathologic or tolerogenic immune response [8]. It has also been observed that the genetically determined deficiency or pharmacological blockade of C3aR/C5aR1 receptors on induced regulatory T-cells (iTreg) increases the production of both murine and human iTreg. Hence, actions targeting the interactions of complement components such as C3a/C3aR and C5a/C5aR1 may facilitate iTreg-mediated tolerance to alloantigens in humans.

## 6. The Relevance of Complement Gene Polymorphism for Kidney Transplant

From a clinical point of view, it is important to maintain homeostasis between the desired effects of complement (the protection against infections and apoptotic cell removal) on one hand and complement-dependent destructive inflammation on the other [99]. It is assumed that complement polymorphisms may affect this balance in transplant recipients. There are functional complement polymorphisms that may impact the functioning of a kidney transplant. Single-gene polymorphisms for MBL, C3, and C4 have been studied in a population of (mainly European) patients after kidney transplantation [103,104,105]. However, these studies did not provide definitive evidence confirming the effect of individual polymorphisms on kidney graft survival or acute rejection. Hence, it seems that single-gene polymorphisms for complement components have only a minimal effect on the transplanted organ. In contrast, the combination of multiple complement component polymorphisms may provide valuable prognostic information after kidney transplantation. This has been confirmed by in vitro studies showing that the combination of C3 polymorphisms and complement factors B and H causes a six-fold increase in complement activation [106]. The inherited set of gene polymorphisms encoding both complement and regulatory proteins is called a complotype. It is believed that the complotype largely determines the individual complement activity [99]. Complotypes that result in a more active complement system could make the kidney allograft more susceptible to rejection-related inflammation, while combinations of polymorphisms causing complement suppression may put the recipient at risk of infection [99]. Because the allograft is capable of synthesizing regulatory proteins as well as C3 and C4 complement components, both membrane-bound and in the liquid phase, donor polymorphisms in these proteins may interact with recipient polymorphisms [100]. A better understanding of the complotypes and the interactions of complement components of donor and recipient can possibly improve the assessment of the risk of acute transplant rejection or graft loss. 

## 7. Conclusions and Prospects for the Future

Complement components play an important role in the pathogenesis of kidney allograft damage. Most studies on the role of complement in the pathogenesis and diagnosis of transplanted kidney function involve humoral rejection. However, complement components and regulatory proteins are also involved in cell-mediated rejection, post-transplant thrombotic microangiopathy, the recurrence of several glomerulopathies in the transplanted kidney, as well as transplant tolerance. Hence, the assessment of complement components can be important in monitoring kidney allograft function in clinical practice. Perhaps such a strategy would allow the early detection of chronic allograft dysfunction, enabling early therapeutic intervention to slow the process and extend the survival of the kidney allograft. Due to an increasingly better understanding of the pathomechanisms of complement involvement in transplanted kidney damage, novel therapies are studied to inhibit its components to improve graft survival. In addition, according to the latest research, analysis of functional complotypes based on the genotype of both the recipient and the donor may, in the future, be a valuable tool for assessing the risk of acute transplant rejection or its loss. For these reasons, further studies are needed to assess the usefulness of complement components in the evaluation of patients after kidney transplantation.

## Figures and Tables

**Table 1:** Recent clinical studies regarding rituximab effects on kidney graft rejection.

Reference	Study Characteristics	Studied Group	Results
Parajuli et al., 2017 [82]	Observational single center study	Kidney transplant recipients with late AMR treated with steroids plus IVIG with (*n* = 40) or without (*n* = 38) rituximab	Rituximab treatment was associated with less frequent graft loss after 1-year follow-up (15% versus 32%).
Tomita et al., 2019 [83]	Propensity-matched retrospective analysis of single center data	Non-sensitized recipients of kidney transplant from AB0-compatible living donors; patients who received rituximab in induction (*n* = 115) were compared with non-rituximab controls (*n* = 115)	Rituximab was associated with lower incidence of biopsy proven acute rejection, lower de novo DSA generation, less frequent CMV infection in 5-year follow-up. No difference in patient and graft survival between groups.
Pathak et al., 2019 [84]	Retrospective single center observational study	Kidney transplant patients given thymoglobulin and low-dose rituximab induction followed by steroid-free immunosuppression (*n* = 1111)	Good patient and graft survival were observed (92.4% and 86.1%, respectively at 12 years), along with low percentage of biopsy-proven acute rejection (12.7%).

**Table 2:** Clinical studies regarding eculizumab in kidney transplant recipients.

Reference	Study Characteristics	Studied Group	Results
Stegall et al., 2011 [85]	Prospectively enrolled patients treated with eculizumab were compared with historical controls	Highly sensitized recipients of kidney graft from living donors with a positive crossmatch; patients treated with plasma exchange protocol plus eculizumab (*n* = 26) were compared with non-eculizumab controls (*n* = 51).	Incidence of AMR during the first 3 months after transplantation was 7.7% in the eculizumab group versus 41.2% in controls.
Kulkarni et al., 2017 [91]	Pilot single center randomized trial	Kidney transplant recipients with chronic persistent DSA and deteriorating renal function, treated with eculizumab for 6 months and then observed for 6 months (*n* = 10) and non-treated (*n* = 5).	During treatment with eculizumab eGFR decrease was slowed as compared to controls. Long-term eculizumab was safe.
Marks et al., 2019 [87]	Phase 2 multicenter randomized open-label trial	Sensitized recipients of living-donor kidney transplant requiring desensitization (*n* = 102): half were treated with eculizumab for 9 weeks post transplantation (*n* = 51); the other half received standard of care.	A composite of biopsy-proven acute AMR, graft loss, death, or loss to follow-up at 9 weeks was observed in 11.8% of the eculizumab group and 29.4% controls. Eculizumab treatment was safe.
Gonzalez Suarez et al., 2019 [86]	Systematic review of observational studies and case series	Adult kidney transplant recipients with aHUS who received eculizumab in treatment or prevention of aHUS recurrence (*n* = 380).	Prophylactic eculizumab was associated with 6.3% (95%CI: 2.8–13.4%) recurrence of TMA and 5.5% (95%CI: 2.9–10.0%) graft loss due to TMA. Treatment with eculizumab was associated with 22.5% (95%CI: 13.6–34.8%) graft loss due to TMA.

**Table 3:** Clinical studies regarding C1-INH in kidney transplant recipients.

Reference	Study Characteristics	Studied Group	Result
Vo et al., 2015 [75]	Phase 1/2 randomized placebo-controlled	Highly sensitized kidney transplant recipients desensitized with IVIG, rituximab with or without plasma exchange (*n* = 20); half received plasma-derived human C1-INH intraoperatively and 7 doses twice weekly (*n* = 10).	Blinded assessment showed reduction in C1q+ HLA antibodies in treatment group, no AMR was observed during the study. Treatment was safe.
Montgomery et al., 2016 [77]	Phase 2b, multicenter double-blind randomized placebo-controlled pilot study	Kidney transplant recipients with biopsy-proved DSA-positive AMR (*n* = 18); half received plasmapheresis and IVIG plus C1-INH every other day for two weeks (*n* = 9), the other half plasmapheresis and IVIG plus placebo.	No difference in renal biopsy on day 20 (primary endpoint). Transplant glomerulopathy was not observed in treatment arm and was observed in 3/7 patients in placebo arm after 6 months. Treatment was safe.
Viglietti et al., 2016 [74]	Prospective, single-arm pilot study	Kidney transplant patients with AMR and acute graft dysfunction nonresponsive to standard therapy; patients were treated with high-dose IVIG plus C1-INH for 6 months (*n* = 6)	eGFR improved during 6-month treatment from mean 38.7 to 45.2 mL/min/1.73 m^2^. C4d deposition in tubular capillaries was less frequent after treatment. Treatment was safe.
Jordan et al., 2018 [101]	Phase 1/2 randomized placebo-controlled single center trial	Recipients of deceased-donor kidney transplant at increased risk for delayed graft function (*n* = 70); half received C1-INH (*n* = 35).	C1-INH did not reduced the need for dialysis at 1-week posttransplant (primary endpoint) but reduced the need for dialysis sessions after 2 to 4 weeks and was associated with better renal function 1 year posttransplant.
Huang et al., 2020 [102]	Post-hoc analysis of randomized placebo-controlled trial of Jordan et al. [101]	Recipients of deceased-donor kidney transplant at increased risk for delayed graft function (*n* = 70); half received C1-INH (*n* = 35).	Cumulative incidence of graft failure was lower over 3.5-year follow-up among patients treated with C1 esterase inhibitor (50 U/kg perioperatively and after 24 h) compared with placebo.
